# Deep Ocean Water Concentrate Changes Physicochemical Characteristics, the Profile of Volatile Components and Consumer Acceptance for Taiwanese Rice Shochu

**DOI:** 10.3390/foods9121806

**Published:** 2020-12-04

**Authors:** Ming-Kuei Shih, Qiao-Yu Hsu, Bo-Kang Liou, Yu-Han Peng, Chih-Yao Hou

**Affiliations:** 1 Graduate Institute of Food Culture and Innovation, National Kaohsiung University of Hospitality and Tourism, Kaohsiung 812, Taiwan; mkshih@mail.nkuht.edu.tw; 2 Department of Seafood Science, National Kaohsiung University of Science and Technology, Kaohsiung 811, Taiwan; tobby426684@gmail.com; 3 Department of Food Science and Technology, Central Taiwan University of Science and Technology, Taichung 406, Taiwan; bkliou6755@ctust.edu.tw; 4 Department of Food Science and Biotechnology, National Chung Hsing University, Taichung 406, Taiwan

**Keywords:** rice shochu, deep-ocean water concentrate, principal component analysis, volatile compounds

## Abstract

To study the effects of deep-ocean water concentrate (DOWC) on sake quality, Taichung No. 10 indica rice (*Oryza sativa* subsp. *indica*) and Tainan No. 11 japonica rice (*O. sativa* subsp. *japonica*) were used as raw materials, and basic physicochemical property parameters in shochu were analyzed differentially. Sake fermentation mash analysis results revealed that DOWC addition did not significantly affect the basic physicochemical properties during sake brewing, but it significantly reduced citric acid and malic acid contents in Taichung No. 10 indica rice sake sample by 52–66% and 73–93%, respectively. DOWC addition significantly increased citric acid content in Tainan No. 11 japonica rice sake sample by 32–202%. Rice shochu analysis results revealed that DOWC addition significantly increased isoamyl acetate, ethyl hexanoate, and ethyl octanoate contents in shochu made from japonica rice and indica rice, respectively. The results indicate that rice variety directly affects the types of volatile compounds in rice shochu. Principal component analysis and sensory evaluation results revealed that DOWC addition affected the composition of volatile compounds in the two types of rice shochu and resulted in differences in flavor evaluation. DOWC addition affects yeast metabolites and directly changes the volatile compound composition and flavor of rice shochu.

## 1. Introduction

Deep ocean water (DOW) is collected at a depth of 300–600 m in the sea and is less contaminated and rich in minerals. DOW has been used in many biotechnology-fermented and functional foodstuffs, and has many health effects [1], such as antiobesity and antidiabetes properties [2], prevention of cardiovascular disease [2], antihypotensive [3], skin protection [4], protection against duodenal ulcers [5], osteoporosis prevention [6], and antifatigue effect [7]. Sake is a Japanese traditional alcoholic beverage typically brewed from rice, water, yeast, and koji. Rice is an essential element in sake brewing. The characteristics of sake are controlled by the choice and combination of rice cultivars. The typical cultivars of shuzo-kotekimai include *Oryza sativa* L. Yamadanishiki, *Oryza sativa* L. Gohyakumangoku, *Oryza sativa* L. Omachi, *Oryza sativa* L. Miyamanishiki, and *Oryza sativa* L. Hyogokitanishiki. In Japan, the top ranked shuzo-kotekimai is Yamadanishiki, especially that grown in Hyogo [8]. Due to the climate, Yamadanishiki cannot be grown in Taiwan. However, Awamori brewed in Japan’s Ryukyu Islands, which has a climate similar to Taiwan, uses rice cultivars that are more suitable for production in tropical regions, and is a close relative of Taiwan’s Indica (*Oryza sativa* subsp. Indica).

Rice proteins are digested into amino acids or peptides by proteases in the koji mold. The released amino acids and peptides create the aroma and taste of brewed sake and are used as major nutrient sources for yeast growth in the sake brewing process. Therefore, it is important to control the digestion and levels of rice protein during the sake brewing process [9]. Analyzing the content and threshold of candidate compounds helps to identify the characteristics of the brewing conditions, such as the type of rice koji used, the type of rice, and the type of shochu. According to Osafune et al. (2020), it can be understood that the characteristic aroma of Awamori was produced when the odor activity value of 1-octen-3-ol was higher in Awamori than in the other categories [10]. Isovaleraldehyde, ethyl caprylate, ethyl caproate, and ethyl 2-methylbutyrate played an important role in imparting the specific odour of rice koji-shochu [11]. In addition, different rice varieties produce unique aroma substances, such as the grassy characteristic aroma perceived in brewed sake made from low-glutelin rice (*Oryza sativa* L. Mizuhonoka) [12]. DOW collected from Kochi prefecture in Japan was used for a study on Japanese sake fermentation, and it was found that DOW increased the levels of aroma components and is related to the biosynthesis of amino acids and fatty acids and the activation of metabolism-related genes [13]. Gogami et al. (2011) analyzed commercially available sakes that were brewed with DOW and found that DOW increased d-amino acid levels in sake, particularly d-Tyr [14]. Mineral contents will affect the metabolic capacity of *Saccharomyces cerevisiae* [15]. Hence, mineral components in DOW stimulate the growth of microorganisms to change sake quality. However, the effects of adding different concentrations of DOW on the fermentation quality of sake brewed from different rice varieties and volatile compounds in rice shochu are still unclear. In addition, the procedure to add concentrations of DOW to adjust the flavor quality of sake is unclear.

Therefore, we used DOW concentrate (DOWC) in Taiwan to brew sake and rice shochu from Taiwan’s unique japonica (*Oryza sativa* subsp. *japonica*) and indica (*Oryza sativa* subsp. *indica*) rice varieties. We hope that a preliminary study on the effects of adding different concentrations of DOW on volatile compound composition of rice shochu products and their correlation with different rice varieties will facilitate future adjustment of sake flavor.

## 2. Materials and Methods

### 2.1. Materials

Taichung No. 10 indica rice and Tainan No. 11 japonica rice (15 kg bags each) were obtained from Yeedon Enterprise Co. Ltd. from Cosco. Deep ocean water concentrate (DOWC) was obtained from Taiwan Yes Deep Ocean Water Co., Ltd. (low sodium product: LC-40K-LS). The relevant ingredients and nutrition labels are shown in Table 1. *Aspergillus oryzae* was purchased from Chinchih brewery. Distilled liquor active dry yeast was purchased from Whole Earth Trading Company. NaCl, acetic acid solution, sodium acetate solution, starch, NaOH, phenol, d-glucose, NH_4_H_2_PO_4_**,** formic acid, and acetonitrile were supplied by Sigma (St. Louis, MO, USA).

### 2.2. Brewing

#### 2.2.1. Rice Koji Making

For self-digestion tests, rice koji was prepared from the two cultivars. Rice grains of 100 g in each cultivar were soaked in water for 1 h and drained for 1 h. These were steamed for 50 min followed by cooling, and the water absorbing ratio of steamed rice was adjusted to 32.0% (*w*/*w*). After the rice was cooled to 38 °C, the rice and 30 mg of conidia (spore) from *Aspergillus oryzae* were mixed in a plastic container. Rice koji making was performed in the container using a temperature and humidity chamber (HIPONT, RH-80, Taichung City, Taiwan). After 8 h, the lid of the container was removed and the surface was then covered with filter paper, while the chamber temperature and humidity were kept at 38 °C and 85%, respectively, for 48 h. Rice koji weight was increased by approximately 13–17%.

#### 2.2.2. Preparation of Shochu

Rice koji and water were mixed at a 1:1.2 ratio and added into the fermentation tank. Commercially available mineral water was used for the control group, and 0.5×, 1×, and 5× DOWC used in the experimental groups were obtained by diluting the original concentration of 60× DOWC with DDW. To the rice koji + water, 0.3% (*w*/*w*) *Saccharomyces cerevisiae* was added and activated using lukewarm water to prepare primary mash. To obtain the starter, the multi-fermentation was implemented for 5 days at 25 °C. To prepare secondary mash, the test rice was steamed and cooled. The further alcohol fermentation was developed by adding the starter to more steamed rice and water, triple and 1.5 times the amount used for the primary mash, respectively. At this point, the ratio of yeast rice and total rice was 25% (*w*/*w*). The mixture was fermented for 9 days at 25 °C, and the pH, total acidity, total sugar, soluble solids, glycerol, and organic acid were analyzed on Days 1, 3, 5, 7, and 9. After fermentation was completed, a stainless steel distillation apparatus was used to obtain shochu from single-batch distillation and distilled until an alcohol by volume (ABV) of 85% was obtained. Following that, the liquor was diluted using distilled water to an ABV of 21%. Approximately 1 kg of the mash was distilled using the steam generated from water in a heated round flask (heated by a mantle heater) after 10 days of fermentation. The distillate was then water-cooled. When the alcohol content in the bundled distillate reached approximately 85%, it was the point of the end of distillation. Next, the distillate was filtered and diluted with deionized water until 21% alcohol was obtained. Shochu samples were then stored at room temperature (25–28 °C) in a dark place before starting GC/MS analysis.

### 2.3. Total Acidity

From the fermentation mash, 5 mL of fermentation solution was centrifuged (8000× *g*, 20 min) and 25 mL of distilled water was added. Following that, titration was carried out using 0.1 N NaOH, and a pH meter (SUNTEX, SP-2500, New Taipei City, Taiwan) was employed for titration to an end point of pH 8.1. Triplicate measurements were performed and the volume of NaOH consumed (in mL) was converted to the acidity of the fermentation mash [16].

### 2.4. Total Sugars

The fermentation mash was centrifuged (8000× *g*, 20 min), and the supernatant was diluted after which 2 mL was collected. About 1 mL of 5% phenol and 5 mL of concentrated sulfuric acid were added to the collected supernatant and cooled in a water bath to room temperature. Spectrophotometry was used to measure the absorbance of the sample at 480 nm, and the total sugar concentration was obtained by calculation using a standard curve [17]. Five different glucose concentrations were prepared, and a standard curve was plotted.

### 2.5. Soluble Solids

The fermentation mash was centrifuged (8000× *g*, 20 min), and the supernatant was collected and used for measurement using a calibrated handheld refractometer (ATAGO, MASTER-BX/S28M, Tokyo, Japan).

### 2.6. pH

The fermentation mash was centrifuged (8000× *g*, 20 min), and the supernatant was collected and measured using a pH meter (SUNTEX, SP-2500, New Taipei City, Taiwan).

### 2.7. Organic Acid Measurement

The method was adapted from Klupczynska et al. (2018) [18]. The fermentation mash was centrifuged (8000× *g*, 20 min), and the supernatant was collected before it was diluted 100 times with DDW. The diluted supernatant was filtered using a 0.22 μm filter and loaded on SHIMADZU LC-MS/MS 8040 for organic acid analysis. The measurement conditions were as follows: mobile phase: pump A: DDW + 0.1% formic acid and pump B: acetonitrile; column: Agela Technologies Venusil XBP C18(2)_4.6 × 150 mm; 3 μm isocratic elution: 5 min; injection volume: 3 μL; *m*/*z* range: 50–300; multiple reaction monitoring (MRM) mode.

### 2.8. Glycerol Measurement

The method was modified from Rainey et al. (2013) [19]. The fermentation mash was centrifuged (8000× *g*, 20 min), and the supernatant was collected before it was diluted 10 times with DDW. The diluted supernatant was filtered using a 0.22 μm filter and loaded on SHIMADZU GC-FID QP2010 for measurement. The measurement conditions were as follows: column: SGE Analytical-54477—30 m × 0.53 mm ID—BP21 0.5 μm; injector temperature: 230 °C; FID temperature: 250 °C; initial temperature: 100 °C that was increased to 250 °C at 40 °C/min, which was maintained for 3 min.

### 2.9. Gas Chromatography-Mass Spectrometry (GC/MS) Volatile Compounds

The method proposed by Yan et al. (2020) was modified [20]. SPME fiber (Supelco, Bellefonte, PA, USA) coated with divinylbenzene/carboxen/polydimethylsiloxane (50/30 μm × 2 cm) was used. The SPME fiber was subjected to impurity removal by applying an injection inlet temperature of 250 °C for 30 min. The samples were preheated to 70 °C. After 30 min, adsorption was carried out for 1 min and the samples were manually injected into the GC/MS (Agilent technologies 7890B) system followed by 5 min of desorption. Splitless measurement was carried out. The chromatography column was as follows: DB-FFAP capillary column (30 m × 0.25 mm × 0.25 µm). Flow rate of the mobile phase gas (helium) was 1 mL/min. Heating condition was as follows: The initial temperature of 50 °C was maintained for 3 min before increasing to 250 °C at a rate of 5 °C/min, which was maintained for 5 min. Injector and transfer line temperature was 250 °C; ion source temperature was 70 eV, 230 °C; quadrupole temperature was 150 °C; and scanning range was 35–550 *m*/*z*. Apart from the unknown peaks that cannot be identified, the other peaks showed the corresponding area, the follow-up comparison of the influence of DOWC addition, and PCA analysis. The mass spectrum of the identified compound was compared with the GC/MS database to confirm the compound type. Each peak identified by MS spectra matching has a corresponding retention time (RT) and Qualitative Analysis (QUAL) comparison rate. The databases used for mass spectrum identification are NIST17 Mass Spectral Library and Agilent Flavor2 database.

### 2.10. Sensory Evaluation

The method of sensory evaluation was modified from Rahayu et al. (2017) [21]. The sample rice shochu was stored at 4 °C until tasting. The sample was placed in a clear glass cup under the regular fluorescent lights. The serving temperature of the wine samples was between 14 and 18 °C, and the room temperature was ~25 °C. One cup (about 10 mL) was tasted for each group and the panelists rinsed by blank drinking sparkling water twice between each group. Taichung rice shochu and Tainan rice shochu were both evaluated based on eight descriptors, including overall, full-bodied, flavor intensity, sweet taste, acidity taste, spicy taste, astringency taste, and taste complexity. In the present study, a sensory-profile analysis with blind testing was conducted by 26 panelists (15 females and 11 males, aged between 21 and 53 years) from National Kaohsiung University of Science and Technology. The majorities of panelists were previously trained on sensory evaluation techniques and had experience of being taste testers. They were asked to evaluate the eight item descriptors using a nine-point scale (0 = not discovered, 1 = slightly discovered, 3 = weak strength, 5 = moderate strength; 7 = strong strength, 9 = very strong strength). The results of the sensory profile analysis were averaged for each sensory attribute and plotted on a spider web diagram.

### 2.11. Statistical Analyses

For all conducted experiments, at least triplicate samples were used. Statistical analysis was performed using XLSTAT 2019 (Addinsoft, New York, NY, USA). All data were analyzed using one-way analysis of variance (ANOVA). Principal component analysis (PCA) was used to reduce the dimensions for evaluating differences in results, and to statistically analyze the volatile compounds through Pearson correlation model [22].

## 3. Results and Discussion

### 3.1. Analysis of the Effects of DOWC Addition on the Physicochemical Characteristics of Fermentation Products

Table 2 shows the final physicochemical measurements of the final fermentation mash (Day 9) of Taichung No. 10 indica rice and Tainan No. 11 japonica rice shochu when different amounts of DOWC were added, which are as follows: (1) pH showed statistical differences, and was the highest in all control groups without DOWC addition and the lowest in the 5× DOWC addition groups. pH was 4.6 and 4.8 and 4.6 and 4.8, respectively; (2) titratable acidity was 323.6–356.4 and 294.8–315.8 mg/L, respectively. The acidity measurement indicates the density of free organic acids such as malic acid, lactic acid, and succinic acid. Sake with high acidity has a sharp and dry taste, whereas that with low acidity has a rich and sweet taste [8] (3) Residual sugar content was approximately 140.2–185.1 and 139.9–179.5 g/L, respectively; (4) total soluble solids (°Brix) content was 20.4–22.9 and 21.3–24.1, respectively; and (5) glycerol content was 9287–10309 and 6789–9372 mg/L, respectively. Compared with Taichung No. 10 indica rice, DOWC addition to Tainan No. 11 japonica rice increased glycerol yield, but its glycerol yield was relatively low. Most non-*Saccharomyces* species showing a reduced ethanol yield produce more glycerol per gram of sugar consumed than wine yeast strains [23,24,25]. However, the glycerol content of sake mash from the two types of rice varieties both exceeded 6000 mg/L. This phenomenon might be owing to the rice varieties used. Glycerol and other polyols are important organoleptic compounds in wine because they contribute to wine mouth feel and body by increasing viscosity. Glycerol content is associated with the TCA cycle in sugar catabolism in yeast. When metabolism is limited, nicotinamide adenine dinucleotide (NADH) accumulates and forms ethanol and glycerol.

With regard to organic acids, DOWC addition significantly reduced citric acid and malic acid contents in Taichung No. 10 rice shochu by 52–66% and 73–93%, respectively. However, citric acid content was increased by 32–302% when DOWC was added to Tainan No. 11 rice. Although there were statistical differences in organic acid contents among the remaining experimental samples, the differences were not particularly prominent. Organic acids, such as malic, succinic, and lactic acids, are crucial factors in determining the taste of Japanese sake. The succinic and malic acids produced by the yeast *Saccharomyces cerevisiae* during sake fermentation confer a refreshing taste to the sake [26]. The majority of sake yeasts predominantly produce succinic acid and relatively little malic acid; therefore, increasing the concentration of malic acid in sake can increase the refreshing characteristic of the beverage [27]. Organic acids are important substrates that contribute to the taste of sake and are mainly produced by yeast (*Saccharomyces cerevisiae*) during fermentation. Succinate is the main taste component produced by yeasts during sake fermentation.

In yeast, malic acid is synthesized via cytosolic and mitochondrial pathways, and the enzymes involved in the malic acid metabolic pathway affect the amount of acid produced. The overexpression of malate dehydrogenase in the reductive TCA cycle of mitochondria increases malic acid production [28]. The glyoxylate pathway enzyme malate synthase and the reductive pathway enzymes pyruvate carboxylase and cytosolic malate dehydrogenase [28] also increase malic acid production. However, overexpression of the malic enzyme, which catalyzes the conversion of malic acid to pyruvic acid in mitochondria, leads to a decrease in malic acid production [29]. Thus, to accurately clarify the organic acid production in sake production, it is necessary to first examine the enzymatic mechanisms that are involved in malic acid production in yeast. Mineral content will affect the metabolic capacity of *Saccharomyces cerevisiae* [15]. A study also pointed out that calcium ion has been reported to affect the fermentation efficiency of *S. cerevisiae* [30]. Therefore, DOWC may affect the metabolism of *S. cerevisiae* in the fermentation mash of indica rice and japonica rice and affect organic acid metabolism to improve sake brewing quality.

### 3.2. Effects of DOWC Addition on Volatile Compound Composition in Rice Shochu

In this study, at least 22 components were found in the identification. We selected 16 volatile components related to sake as the main indicator components. Follow-up comparison of the influence of DOWC addition and PCA analysis. Table 3 shows the GC/MS analysis of volatile compounds in rice shochu, which are divided into three major classes: aldehydes, alcohols, and esters. Rice shochu analysis revealed that DOWC addition significantly increases isoamyl acetate, ethyl hexanoate, and ethyl octanoate contents in rice shochu made from japonica rice and indica rice. Isoamyl acetate content was 26–79% and 24–70%, respectively; ethyl hexanoate content was 38–165% and 90–194%, respectively; and ethyl octanoate content was 11–115% and 40–70%, respectively. In addition, DOWC addition significantly increased ethyl acetate content by 166–263% in Tainan No. 11 rice shochu but by a maximum of only 113% in Taichung No. 10 rice shochu. These volatile compounds can also be detected in shochu products [11].

Isoamyl acetate and ethyl acetate are aroma indicator components of Ginjo-sake [31]. In addition, there have been some reports that ethyl hexanoate, which results in a fruity aroma, is a key compound in beer and sake. Furthermore, ethyl acetate, isobutanol, isoamyl acetate, isoamyl alcohol, and ethyl hexanoate are the five major compounds responsible for the orthonasal aroma of sake [32]. The indica rice and japonica rice used in this study have a rice polishing ratio of 60% and 55%, respectively. The higher the rice polishing ratio, the lower the residual bran content, the higher the level of protein that can be used by microorganisms, and the higher the fatty acid content. Ginjo-shu is a type of sake that is brewed using highly polished rice with a polishing ratio of 60% or lower, for long periods at low temperatures. Ginjo-shu is characterized by a fruity flavor, referred to as ginjo-ko, which is mainly derived from isoamyl acetate and ethyl caproate. Isoamyl acetate is synthesized from acetyl-CoA and isoamyl alcohol by alcohol acetyltransferase (AATase, EC 2.3.1.84), which is encoded by ATF1 or ATF2 in yeast [33,34,35]. Studies on ATF1/ATF2 deletion mutants have revealed that Atf1p plays a major role in isoamyl acetate production in the brewing processes of beer [36]. However, ATF1 expression is inhibited by unsaturated fatty acids [37,38]. Specifically, an 18-bp fragment that encodes the Rap1p-binding domain of the 5′-flanking region of ATF1 is crucial for transcriptional regulation of the gene by unsaturated fatty acids [39]. In other words, in sake brewing using polished rice with a high polishing ratio, isoamyl acetate production by *Saccharomyces cerevisiae* markedly decreases because ATF1 expression is repressed by unsaturated fatty acids derived from the outer layer of rice.

Although DOWC addition will change the composition of volatile compounds, this generation of certain volatile compounds and whether this phenomenon is dependent on the concentration of DOWC added actually differ depending on whether indica rice or japonica rice is used. For example, DOWC addition dramatically increased ethyl laurate in Taichung No. 10 indica rice by 40.0–82.2 folds. However, there was no significant difference in ethyl laurate content between the control group and experimental group in Tainan No. 11 japonica rice shochu. In addition, different *Aspergillus oryzae* strains produce different aroma characteristics. Any factors that affect *A. oryzae* metabolism change the flavor characteristics of the product. In this study, *A. oryzae*, which is commonly used by the Taiwanese people, and distilled liquor active dry yeast were used, and the rice varieties used were Taiwanese varieties. Therefore, the volatile compounds’ composition differs from the results of previous studies. For example, 2-methylbenzaldehyde only appeared in Tainan No. 11 japonica rice shochu, whereas ethenyl formate and ethyl heptanoate were only present in Taichung No. 10 indica rice shochu, and these were not related to DOWC addition. However, 2-methylbenzaldehyde, ethenyl formate did not appear in other studies on sake, whereas ethyl heptanoate appeared in the aroma substance of brown sugar shochu [40]. Interestingly, ethyl heptanoate was almost not seen in rice shochu studies. This phenomenon suggests that the production of this class of volatile compounds is not related to DOW addition and should be related to rice variety, and *A. oryzae* and yeast strains. Analysis of alcohol compounds found that there were no major differences in isobutanol and isoamyl alcohol contents between Taichung No. 10 rice shochu and Tainan No. 11 rice shochu.

Yoshizaki, et al. (2010) presented that the volatile compounds from white and black koji prepared by *A. luchuensis mut. kawachii* and *A. luchuensis*, respectively, are distinct from those of yellow koji prepared by *A. oryzae* [41]. Rahayu et al. (2016) analyzed critical aroma substances in shochu fermented from red yeast rice and sensory evaluation showed that red koji-shochu has the distinctive flavors of cheese, sour, milky, and oily [21]. In addition, some studies conducted with respect to the direct or indirect effects of koji on shochu flavor proved that koji itself showed characteristic mushroom or chestnut flavors distinct from those of rice as a material. Ito et al. (1990) [42] identified 16 types of alcohols, ketones, and aldehydes and an ester in koji, and it was demonstrated that the growth stage of koji mold affects the composition of these volatile compounds [37,38]. The type of species used for preparation of koji’s odor has also been shown. From the above results, it can be seen that DOWC addition changes volatile compound contents in rice shochu and has the strongest effect on ester volatile compounds.

### 3.3. Principal Component Analysis of Volatile Compounds in Rice Shochu

**Figure 1:**
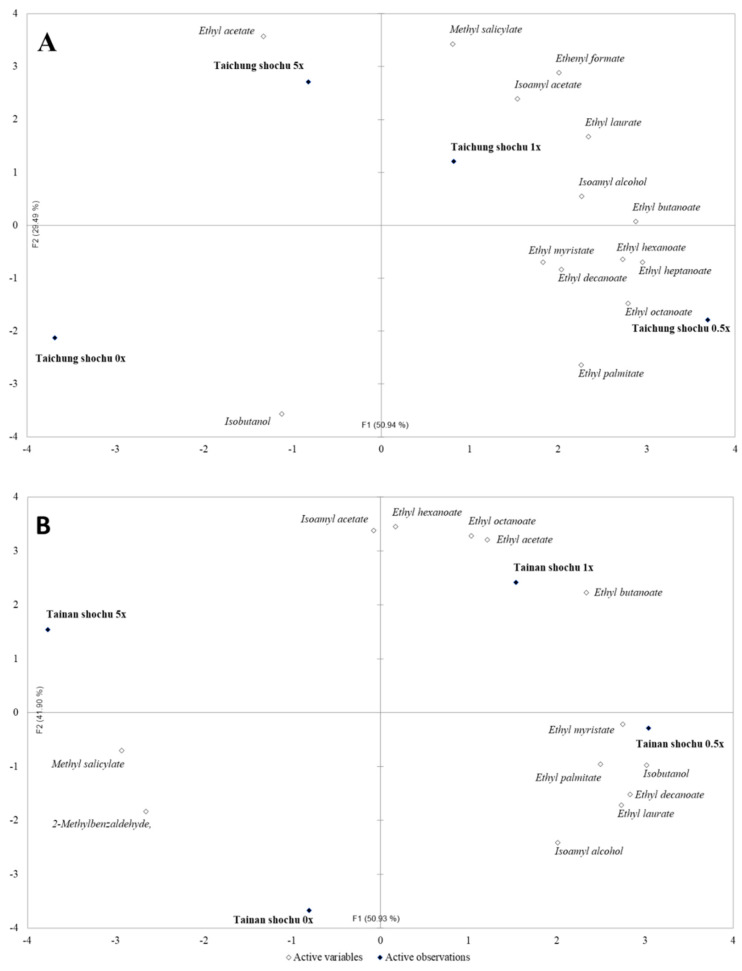
A bi-plot by principal component analysis of volatile compounds for the Taiwanese rice shochu samples with different DOWC concentration selected by GC/MS. (**A**) Taichung No. 10 rice shochu; (**B**) Tainan No. 11 rice shochu.

Principal component analysis was carried out to understand changes in volatile compounds in rice shochu samples made from different rice varieties when different concentrations of DOWC were added. Figure 1A,B represent the principal component analysis of volatile compounds in Taichung No. 10 and Tainan No. 11 rice shochu samples. It can be seen that F1 + F2 both explained 80.43% and 92.83% of variation, respectively. In the drawing results of the PCA analysis, the rich alcohol statistics data were removed to increase the contribution rate of other volatile components. As DOWC concentration increased, the combination of volatile/aroma components in rice shochu changed. Figure 1A shows that the control group (without DOWC(0×)) in Taichung No. 10 rice shochu only correlated with isobutanol. When 0.5× and 1× DOWC was added, there were many kinds of volatile substances. When DOWC was added up to 5×, volatile substances disappeared and only ethyl acetate remained. The result appeared to be the same in Tainan No. 11 rice shochu (see Figure 1B). The result in the control group (DOWC (0×)) shows correlation with methyl salicylate, 2-methylbenzaldehyde; when 0.5× and 1× DOWC was added, many kinds of volatile substances appeared; and when DOWC was added up to 5×, volatile substances disappeared and only isoamyl acetate remained. In addition, in Taichung No. 10 rice shochu, the 0.5× DOWC addition appeared to be mainly related to ester volatile compounds, such as ethyl myristate, ethyl hexanoate, ethyl decanoate, ethyl heptanoate, ethyl octanoate, and ethyl palmitate. In Tainan No. 11 rice shochu, the relation appeared to be with alcohols and esters, such as isobutanol, isoamyl alcohol, ethyl myristate, ethyl palmitate, ethyl decanoate, and ethyl laurate.

To sum up, DOWC showed a dose effect on the production of volatile/aroma components in rice shochu, and different rice wine (made with different rice varieties) showed different composition combinations. The findings showed that DOWC might have affected the metabolic properties of microbial winemaking [13,15,30], and different rice ingredients had their own unique flavors [12]. If DOWC affects the decomposition of rice protein by microorganisms, it means that different amino acids and peptide metabolites will be further produced during the wine brewing process [9,14], which will affect the final flavor characteristics of the sample. As a result, DOWC has created a combination of different volatile/aromatic compounds in both Taichung No. 10 rice shochu and Tainan No. 11 rice shochu.

Interestingly, even though the 5× DOWC reduced the richness of rice shochu in the combination of volatile/aroma components, it can be seen that the addition samples of DOWC strengthened the relatively high correlation with ethyl acetate (rice aroma) and Isoamyl actate (Ginjo-shu) in Taichung No. 10 rice shochu and Tainan No. 11 rice shochu, respectively. Isoamyl acetate and ethyl acetate are aroma indicator components of Ginjo-sake [31]. By combining Figure 1A and Figure 1B, it can be seen that in addition to changing the volatile compound composition in rice shochu samples when DOWC was added, the effects of DOWC on the rice shochu product were also affected by the rice variety. Therefore, based on the PCA analysis results, we can suggest that conducting sample brewing and analysis in the 1×–5× DOWC addition can obtain a balanced aroma. On the whole, DOWC addition can regularly change the composition of volatile/aromatic compounds, and it is possible to find a more balanced aroma characteristic by adjusting the DOWC concentration.

### 3.4. Sensory Evaluation

**Figure 2:**
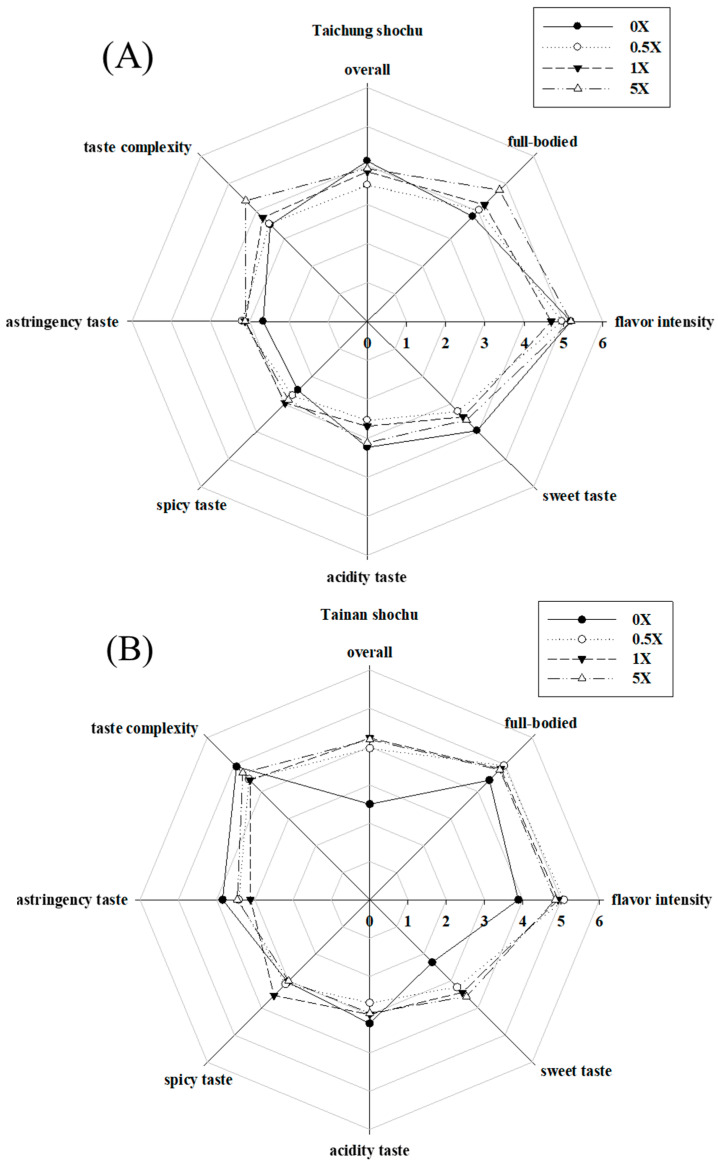
Sensory evaluation of rice shochu sample. Each value was expressed as the mean of evaluation scores (*n* = 26). The results of Taichung No. 10 rice shochu (**A**) and Tainan No. 11 rice shochu (**B**) were brewed with DOWC with different concentrations.

To further understand the effects of DOWC addition on the flavor of rice shochu samples, we conducted acceptability analysis by sensory evaluation using a 9-point hedonic method for overall liking degree, intensity, aroma, sweetness, sourness, spiciness, astringency, and complexity. The results revealed that Taichung No. 10 added with 5× DOWC had the most significant scores for aroma, complexity, and intensity, whereas the control group had the highest scores for sweetness, sourness, and overall liking degree (Figure 2A). The Tainan No. 11 rice shochu (Figure 2B) control group had higher scores for liking degree, sweetness, and aroma than Taichung No. 10. DOWC addition changed the organic acid content in sake and aroma compound composition in shochu from indica rice and japonica rice and further changed the flavor and taste evaluation results of the samples.

## 4. Conclusions

In this study, we selected Taichung No. 10 indica rice and Tainan No. 11 japonica rice that were produced in Taiwan and DOWC to carry out a preliminary study to evaluate the possibility of developing a home-grown rice shochu of Taiwan. In addition to gas chromatography-mass spectrometry analysis of rice shochu made from the two rice varieties in which DOWC was added, which showed that there were 16 major aroma substances, such as alcohols, esters, and aldehydes, our PCA data also support the following results: DOWC addition can change the volatile compound composition of rice shochu samples, but the effects of DOWC on rice shochu products also differ between rice varieties. For example, DOWC addition can regularly change the composition of volatile/aromatic compounds, and it is possible to develop a more balanced aroma characteristic by adjusting the DOWC concentration. In the future, the possible mechanisms by which enzyme activity and metabolites change the composition of volatile compounds in rice shochu can be examined while evaluating how DOWC affects yeast metabolism to examine the application of DOWC in adjusting sake flavor.

## Figures and Tables

**Table 1:** Nutrient composition of deep ocean water and rice raw materials.

	Contents				Amounts per 100 g							Polish Ratio
Nutrient Content	Mg	Ca	Na	K	Calories	Protein	Total Fat	Carbohydrates	Ash	Dietary Fiber	Water	-
**Mineral water ^1^**	1.5~16.4 mg/L	3.0~40.2 mg/L	1.5~14.4 mg/L	- ^5^	-	-	-	-	-	-	-	-
**Deep ocean water concentrate (DOWC) ^2^**	>50,000 mg/L	<500 mg/L	<5000 mg/L	>4000 mg/L	-	-	-	-	-	-	-	-
**Taichung No. 10 ^3^**	19 mg/100 g	5 mg/100 g	2 mg/100 g	85 mg/100 g	357 kcal	7.4 g	0.7 g	78.5 g	0.4 g	0.6 g	16.0 g	60~61%
**Tainan No. 11 ^4^**	4 mg/100 g	4 mg/100 g	2 mg/100 g	71 mg/100 g	347 kcal	6.7 g	0.5 g	77.4 g	0.2 g	0.8 g	15.4 g	55~56%

^1^ General commercial mineral water. ^2^ The product specification is 60-fold deep sea concentrated water (LC-40K-LS). ^3^ Taichung No. 10 indica rice (*Oryza sativa* subsp. *indica*). The nutritional content was retrieved from the Taiwan FDA Food Nutrition Database. ^4^ Tainan No. 11 japonica rice (*O. sativa* subsp. *japonica*). The nutritional content was retrieved from the Taiwan FDA Food Nutrition Database. ^5^ No data form mineral water database.

**Table 2:** Analysis of physicochemical characteristics as well as glycerol and organic acid contents in fermentation mash on Day 9 of fermentation.

Rice Varieties	pH	Titratable Acidity (mg/L)	Residual Sugar (g/L)	Total Soluble Solids (°Brix)	Glycerol (mg/L)	Lactic Acid (mg/L)	Citric Acid (mg/L)	Malic Acid (mg/L)	Succinate Acid (mg/L)
**Taichung No. 10**									
0× *	4.8 ± 0.0 ^a^	327.7 ± 6.0 ^ab^	175.5 ± 11.4 ^b^	22.8 ± 0.1 ^b^	9287 ± 139 ^b^	100.3 ± 3.8 ^c^	274.1 ± 28.6 ^a^	302.8 ± 12.9 ^a^	1671.4 ± 13.6 ^a^
**0.5×**	4.8 ± 0.0 ^b^	335.9 ± 6.0 ^b^	173.2 ± 7.7 ^b^	22.5 ± 0.1 ^c^	10070 ± 157 ^a^	153.2 ± 3.1 ^a^	92.5 ± 2.9 ^c^	83.4 ± 1.4 ^b^	1700.6 ± 12.8 ^a^
**1×**	4.7 ± 0.0 ^c^	323.6 ± 6.0 ^c^	185.1 ± 3.7 ^a^	22.9 ± 0.0 ^a^	9549 ± 60 ^b^	111.9 ± 6.2 ^b^	122.7 ± 1.1 ^bc^	21.4 ± 0.3 ^d^	826.2 ± 23.0 ^c^
**5×**	4.6 ± 0.0 ^d^	356.4 ± 0.0 ^a^	140.2 ± 2.3 ^c^	20.4 ± 0.1 ^d^	10309 ± 87 ^a^	108.3 ± 3.2 ^bc^	131.4 ± 1.5 ^b^	54.0 ± 0.4 ^c^	1347.2 ± 3.9 ^b^
**Tainan No. 11**									
**0×**	4.8 ± 0.0 ^a^	294.8 ± 6.0 ^b^	139.9 ± 19.3 ^c^	21.4 ± 0.0 ^c^	6789 ± 208 ^c^	186.7 ± 4.9 ^c^	68.0 ± 1.7 ^d^	248.7 ± 10.9 ^b^	1123.4 ± 23.3 ^b^
**0.5×**	4.7 ± 0.0 ^c^	315.8 ± 0.0 ^a^	170.5 ± 12.8 ^ab^	24.1 ± 0.1 ^a^	6870. ± 40 ^c^	310.8 ± 8.8 ^b^	89.6 ± 1.9 ^c^	251.7 ± 5.6 ^b^	1191.3 ± 12.6 ^b^
**1×**	4.8 ± 0.0 ^b^	315.8 ± 0.0 ^a^	160.9 ± 6.3 ^b^	21.3 ± 0.1 ^c^	9372 ± 152 ^a^	102.8 ± 3.9 ^d^	196.1 ± 3.5 ^b^	304.0 ± 10.7 ^a^	1313.1 ± 17.2 ^a^
**5×**	4.6 ± 0.0 ^d^	315.8 ± 0.0 ^a^	179.5 ± 16.1 ^a^	23.5 ± 0.0 ^b^	7814 ± 186 ^b^	326.8 ± 13.0 ^a^	205.4 ± 3.1 ^a^	122.1 ± 3.0 ^d^	977.0 ± 5.9 ^d^

* Dilution folds of original deep ocean water concentrates. Data expressed as means ± SDs (*n* = 3). Values with different letters are specific to each rice variety and show significant differences (ANOVA, LSD test, *p* < 0.05).

**Table 3:** Analysis of volatile compound composition in Taichung No. 10 rice shochu and Tainan No. 11 rice shochu after DOWC addition.

Compound ^1^	Area (%) ^2^							
	Taichung No. 10 Rice Shochu ^3^				Tainan No. 11 Rice Shochu ^4^			
	0×	0.5×	1×	5×	0×	0.5×	1×	5×
**Aldehydes**								
**2-Methylbenzaldehyde**	N/D ^5^	N/D	N/D	N/D	0.177	0.104	0.110	0.165
**Alcohols**								
**Ethanol**	68.307	60.705	63.643	58.587	67.529	65.293	64.393	77.516
**Isobutanol**	3.774	3.578	3.296	3.342	3.583	4.7094	3.655	2.109
**Isoamyl alcohol**	14.859	16.740	15.214	16.256	19.152	15.5716	13.921	5.250
**Esters**								
**Ethenyl formate**	1.008	1.401	1.390	1.511	N/D	N/D	N/D	N/D
**Ethyl acetate**	2.128	0.486	3.373	4.529	1.327	3.963	4.822	3.525
**Ethyl butanoate**	0.407	0.782	0.559	0.594	0.464	0.593	0.581	0.509
**Isoamyl acetate**	6.278	9.794	7.912	11.248	4.750	5.867	8.063	7.083
**Ethyl hexanoate**	0.757	2.006	1.044	1.230	0.418	0.796	1.229	1.008
**Ethyl heptanoate**	0.050	0.092	0.076	0.058	N/D	N/D	N/D	N/D
**Ethyl octanoate**	1.389	2.988	1.860	1.535	1.175	1.672	1.997	1.649
**Ethyl decanoate**	0.474	0.656	0.722	0.402	0.461	0.558	0.420	0.249
**Methyl salicylate**	0.439	0.482	0.617	0.569	0.659	0.544	0.493	0.768
**Ethyl laurate**	0.001	0.076	0.108	0.052	0.059	0.072	0.050	0.026
**Ethyl myristate**	0.055	0.085	0.105	0.039	0.037	0.060	0.041	0.031
**Ethyl palmitate**	0.074	0.129	0.082	0.049	0.211	0.199	0.226	0.111

^1^ Volatile compounds detected in rice shochu. ^2^ Area (%) expresses volatile compounds content by GC/MS. ^3^ Taichung No. 10 rice shochu was brewed with DOWC with different concentrations. ^4^ Tainan No. 11 rice shochu was brewed with DOWC with different concentrations. ^5^ Not detected in sample.
